# Nanocellulose-Based Carbon Aerogel Loaded with Composite Metal Oxides and Its Fenton Catalytic Oxidation Degradation of Phenol

**DOI:** 10.3390/nano15161292

**Published:** 2025-08-21

**Authors:** Yunpeng Gao, Jinyang Chen

**Affiliations:** School of Environmental and Chemical Engineering, Shanghai University, Shanghai 200444, China; gaoyunpeng@shu.edu.cn

**Keywords:** Fenton oxidation, carbon aerogel, phenol, degradation, complex metal oxide

## Abstract

The development of stable and efficient heterogeneous Fenton oxidation for organic pollutant degradation is crucial to avoid iron sludge formation and cumbersome filtration processes. In this study, iron oxide/carbon aerogel was prepared via the sol–gel method, freeze-drying, and high-temperature carbonization using iron nitrate heptahydrate, ammonium hydroxide, and cellulose as raw materials, with polyvinylimine serving as the crosslinking agent. To enhance the pH adaptability of the catalyst, copper and cerium elements were introduced. The characterization results demonstrate the iron (III) oxide within the carbon aerogel, achieving phenol degradation efficiency exceeding 95% within 120 min. Meanwhile, the introduction of copper and cerium accelerated the degradation of phenol while maintaining a certain catalytic degradation effect at pH 5-7. In addition, the catalyst exhibited excellent recyclability, retaining 85% of its initial degradation efficiency after five reaction cycles. This work offers a new method for the development of heterogeneous Fenton catalysts.

## 1. Introduction

Phenol, an essential chemical raw material, finds extensive applications in medicine and chemical synthesis, among other fields [1]. However, its degradation difficulty and toxicity make phenolic wastewater a threat to human health [2] through potential skin damage, respiratory irritation, and carcinogenic effects [3]. Advanced oxidation technologies (AOPs) have emerged as effective treatment methods that utilize physical, chemical, or biological approaches to generate highly reactive oxidative radicals for organic pollutant degradation [4]. Among these, Fenton oxidation stands out as a prominent AOP technology that efficiently decomposes phenol compounds through hydroxyl radicals (·OH) generation via Fe^2+^-catalyzed H_2_O_2_ decomposition [5]. However, the Fenton process exhibits significant limitations, operating effectively only within a narrow acidic pH range (2–4) and generating iron-containing sludge that reduces the catalytic efficiency while presenting challenges in filtration and separation [6].

In recent years, ion-supported solid Fenton oxidation catalysts have emerged as promising materials [7], demonstrating reusability through straightforward solid–liquid separation [8]. Feng et al. constructed the CuFe_2_O_4_/H_2_O_2_/AA/photo-Fenton system [9], and under optimal conditions, its degradation rate of phenol reached 98.6%. However, after four cycles, this decreased to 74%. Xia et al. constructed a dendritic Fe-Cu bimetallic catalyst composed of a Cu/Fe_3_O_4_ shell and a FeCu core [10]. The presence of Cu broadened the pH range of the Fenton reaction, and it was active at a pH of 4. However, despite there being a significant decline in activity at pH 6, its material could hardly adsorb phenol and could not pre-enrich pollutants. Zeng et al. synthesized magnetic carbon microspheres [11] with easy recyclability and good cyclability, still maintaining over 85% degradation efficiency after six cycles. However, they were severely limited by pH. The degradation efficiency was greatly restricted at a pH of 4, and at pH 6, this dropped to about 10%. The material we synthesized still had a relatively high degradation rate after five cycles. The presence of nanofiber carbon aerogel is conducive to the pre-enrichment of phenol, and the introduction of Cu broadens the material’s adaptability to pH, while the iron element exists in the form of iron(III) oxide, endowing the material with certain magnetism and ease of recycling.

Herein, we fabricated an iron oxide-loaded nanocellulose-based carbon aerogel through a one-pot sol–gel method [12]. Cellulose, serving as a stable carbon-rich precursor, offers the advantages of abundance and wide availability [13]. The derived nanocellulose–carbon aerogel exhibits excellent stability, an electron-rich structure, and superior porous characteristics [14]. During synthesis, iron ion salts, ammonia solution, and crosslinking agents were fully dispersed into the cellulose solution [15]. Following thorough mixing and dispersion at room temperature, hydrogel formation was achieved [16], and subsequent freeze-drying and vacuum carbonization yielded the iron oxide-incorporated cellulose-based carbon aerogel [17]. To further broaden the Fenton reaction’s pH applicability, copper and cerium ions were introduced to synthesize a polymetal oxide/cellulose-based carbon aerogel [18]. Meanwhile, the material’s catalytic degradation efficiency, recyclability, and phenol degradation mechanisms were systematically investigated [19].

## 2. Materials and Methods

### 2.1. Materials

Nanocellulose was obtained from the Zhejiang Transfar Group (Hangzhou, China), while we purchased the following purchased from Sinopharm Chemical Reagent Co., Ltd. (Shanghai, China): iron(III) nitrate nonahydrate (AR, ≥98.5%), copper(II) nitrate trihydrate (AR, 99.0–102.0%), cerium(III) nitrate hexahydrate (AR, 99.5%), phenol (AR, ≥99.0%), hydrogen peroxide (30% aqueous solution, AR, ≥30.0%), sodium hydroxide (AR, ≥96.0%), nitric acid (AR, 65.0–68.0%), potassium ferricyanide (CP, ≥99.0%), 4-aminoantipyrine (AR, ≥98.5%), ammonium chloride (GR, ≥99.8%), and ammonia water (20%). Polyethyleneimine (99%, MW 600) was procured from RON’s Reagent (Shanghai, China), and deionized water was prepared in the laboratory.

### 2.2. Preparation of Metal Oxide/Carbon Aerogel

**Figure 1:**
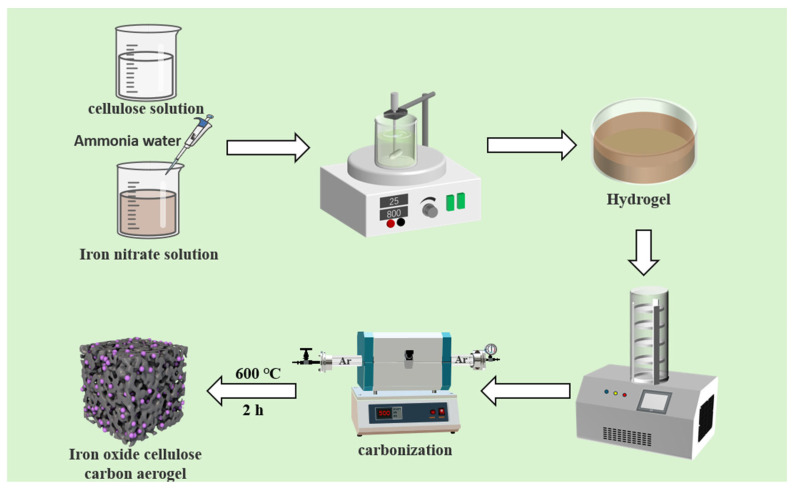
Schematic diagram of the carbon aerogel synthesis.

Figure 1 illustrates the synthesis procedure for metal oxide/carbon aerogel. Initially, 2 g of nonahydrate ferric nitrate was dissolved in 100 mL of deionized water under magnetic stirring, followed by the addition of 3 mL of ammonia water (20%) with continued magnetic stirring (800 r/min) for 1 h to ensure complete dispersion of the formed ferric hydroxide. Further, 2 g of nanocellulose was dispersed in 100 mL deionized water through an ultrasonic treatment (SONICS-1800W, Taixing Xingjian Chemical Machinery Co., Ltd., Taixing, China) for 10 min to obtain a 2 wt% nanocellulose solution. The nanocellulose solution was then added to the ferric hydroxide solution along with 2 mL polyethyleneimine, and the mixture was subjected to further magnetic stirring for 2 h. 

The homogeneous dispersion was poured into multiple cylindrical molds (25 mm diameter × 15 mm height) to form the hydrogels, which were subsequently freeze-dried for 48 h using a lyophilizer (SCIENTZ-18ND, Ningbo Xinzhi Biotechnology Co., Ltd., Ningbo, China) to obtain aerogels. The resulting aerogels were then subjected to carbonization in a tube furnace (SK-G04123K, Tianjin Zhonghuan Electric Furnace Co., Ltd., Tianjin, China) under an argon atmosphere with a heating rate of 3 °C/min to 600 °C, followed by a 2 h isothermal hold, yielding the final iron oxide/cellulose-based carbon aerogel.

The synthesis of iron oxide/copper oxide carbon aerogel followed an analogous procedure, wherein 2 g of nonahydrate ferric nitrate and 0.3 g of trihydrate copper nitrate were dissolved in 100 mL deionized water, followed by the addition of 4.5 mL ammonia water under magnetic stirring (800 r/min) for 1 h. For the iron oxide/copper oxide/cerium oxide carbon aerogel, 2 g nonahydrate ferric nitrate, 0.3 g trihydrate copper nitrate, and 0.2 g cerium nitrate hexahydrate were dissolved in 100 mL water, prior to the addition of 4.5 mL ammonia water and subsequent magnetic stirring (800 r/min) for 1 h. The resulting materials were designated as Fe_3_O_4_/CA, Fe_3_O_4_/CuXO/CA, and Fe_3_O_4_/CuXO/CeXOY/CA for the iron oxide/cellulose-based carbon aerogel, the iron oxide/copper oxide carbon aerogel, and the iron oxide/copper oxide/cerium oxide carbon aerogel, respectively.

### 2.3. Characterization

The XRD patterns of the samples were obtained on an X-ray diffractometer (Rigaku SmartLab SE, Tokyo, Japan) with a step size of 0.02°, scanning rate of 2°/min, and scanning range of 5–90°. The SEM and EDS images of the samples were captured using a 300 scanning electron microscope (GeminiSEM 300,German ZEISS, Oberkochen, Germany) equipped with an SE2 secondary electron detector, operating at 3 kV for morphology shooting and 15 kV for energy spectrum mapping shooting. The XPS spectra were carried out on a Thermo Scientific K-Alpha (Waltham, MA, USA) with a 400 μm spot size, 12 kV working voltage, and 6 mA filament current. The full-spectrum scanning energy was 150 eV (1 eV step), and the narrow-band scanning passability was 50 eV (0.1 eV step). The nitrogen adsorption and desorption tests were conducted using a Micromeritics ASAP 2460 analyzer (Norcross, GA, USA) at a liquid nitrogen temperature of 77 K. The hysteresis loop was measured using a Quantum Design PPMS DynaCool (San Diego, CA, USA).

### 2.4. Adsorption of Phenol Aqueous

**Figure 2:**
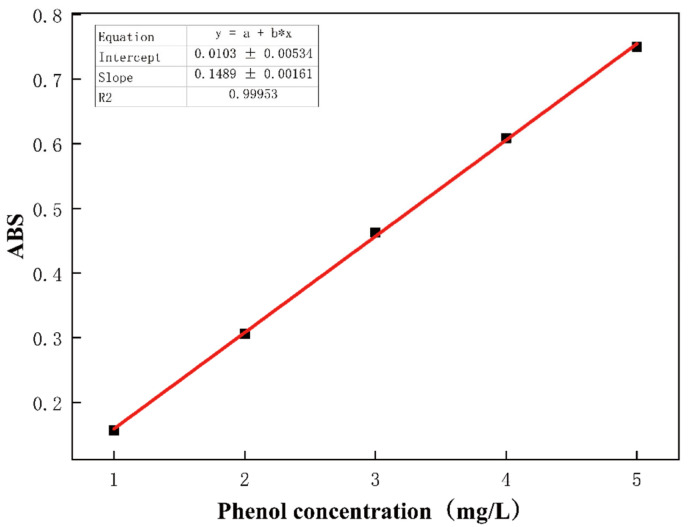
The standard curve of phenol concentration measured by the tetraminoantipyrine method.

Phenol was selected as a typical phenolic substance to evaluate the adsorption performance of carbon aerogel. A predetermined quantity of carbon aerogel was introduced into a 50 mL conical flask containing 40 mL of phenol solution (100 mg/L). Prior to adsorbent addition, the initial pH of the solution was adjusted to the desired value using 0.1 M HCL and 0.1 M NaOH solutions. The mixture was then agitated in a constant temperature shaker at 250 rpm reaching the predetermined contact time was reached. Subsequently, the sample was centrifuged at 4000 r/min for 10 min to separate the catalyst from the solution. The supernatant was carefully taken after centrifugation, and the phenol concentration was determined by the 4-aminoantipyrine method. The absorbance was measured at 510 nm with a UV spectrophotometer (WF Z UV-2800H, Unico, Suite E, Dayton, NJ, USA), and the phenol concentration was calculated by referencing a pre-established standard calibration curve (Figure 2). The adsorption capacity was calculated according to Formula (1):

(1) $$Q_{e}=\frac{\left(C_{0}-C_{t}\right)V}{m}$$

Here, C_0_ (mg/L) represents the initial phenol concentration, while C_t_ (mg/L) corresponds to the phenol concentration after 30 min of reaction; V (mL) refers to the solution volume, and m (mg) represents the mass of the carbon aerogel.

We then dispersed a certain mass of adsorbent (10 mg) into 20 mL phenol solutions of different concentrations (50, 60, 70, 80, 90, and 100 mg/L), adjusted the initial pH of the solution to 3, and shook this at 250 rpm using a constant-temperature shaker at different temperatures (298, 308, and 318 K) until the reaction reached equilibrium. Next, we measured the remaining phenol concentration in the supernatant and calculated the adsorption capacity of phenol through Formula (1).

### 2.5. The Catalytic Degradation of Phenol with Metal Oxide/Carbon Aerogel Materials

The metal oxide/carbon aerogel material was added to the phenol aqueous solution, followed by the addition of a predetermined amount of H_2_O_2_ to initiate the Fenton catalytic oxidation reaction (phenol concentration: 100 mg/L, catalyst dosage: 0.5 g/L, PH: 3.0, H_2_O_2_ concentration: 3.6 mM, temperature: 298 K). Phenol concentration during the reaction was monitored using a UV spectrophotometer after filtration. The catalytic degradation rate (%) was obtained by calculating the concentration change in phenol at a wavelength of 510 nm.

(2) $$Degradation\;rate\left(\%\right)=\frac{{(C}_{0}-C_{t})}{C_{0}}\times 100$$

C_0_ and C_t_ are the phenol concentrations at the initial and reaction time t, respectively.

For the recycling experiment, the catalyst was recovered upon reaction completion via centrifugation (4000 r/min), subsequently transferred into a methanol solution containing 5% (*v*/*v*) acetic acid, subjected to multiple washing times, and finally dried at 353 K.

### 2.6. Radical Inhibitors Experiment

We took five 300 mL beakers and added 200 mL of phenol solution (phenol solution: 100 mg/L) to each of them. The first and second beakers were the blank group, with the first beaker only containing phenolicquinone (BQ, 5 mM) and the second beaker only isopropanol (IPA, 40 mM). The third and fourth beakers comprised the quenching group, and 0.5g/L of catalyst was added to each of them. The third beaker contained phenolicquinone, and the fourth beaker isopropanol. The fifth beaker represented the control group, only containing phenol solution and a catalyst. We adjusted the pH of all five beakers to 3.0 and added H_2_O_2_ to start the experiment. The reaction proceeded in the dark to avoid the influence of light on free radicals. After the reaction was initiated, the beakers were stirred at a constant temperature (25℃), after which 5ml of the samples were collected every ten minutes, immediately adding 0.1ml of methanol to terminate the reaction. Finally, we measured the concentration of phenol using a spectrophotometer.

### 2.7. Fukui Function Calculation

The molecular structure of phenol was plotted using GaussView 6.0 and preliminarily optimized. The electronic structure and Fukui index were calculated using NI Multiwfn 14.3 quantum chemical analysis software.

## 3. Results and Discussion

### 3.1. Morphology

**Figure 3:**
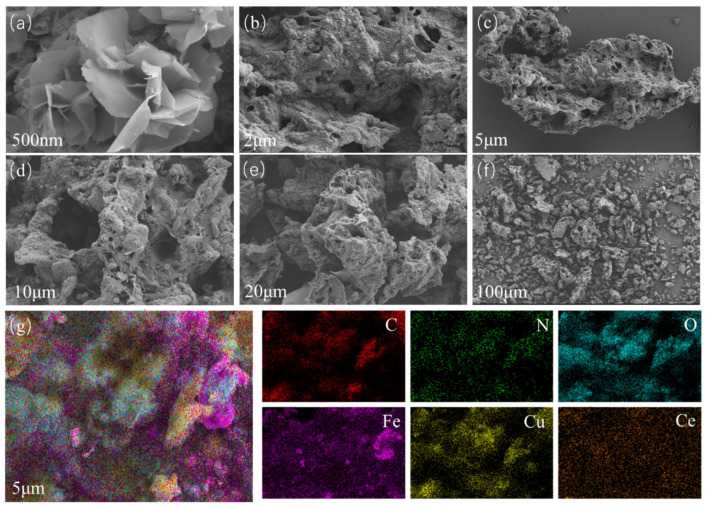
(**a**–**f**) SEM images of metal oxide/carbon aerogels at different magnifications. (**g**) The EDX spectrum of the sample and the distribution maps of each element.

In this section, we present the surface morphology of the prepared composite metal oxide-based carbon aerogel. As shown in Figure 3, the material prepared by crosslinking cellulose with polyethyleneimine had a porous structure and a rough surface, which is conducive to the adsorption and degradation of phenol. Moreover, SEM images (a) and (b) show Fe_3_O_4_/CA, (c) and (d) are of Fe_3_O_4_/Cu_X_O/CA, and (e) and (f) illustrate Fe_3_O_4_/Cu_X_O/Ce_X_O_Y_/CA. Meanwhile, EDS confirms the uniform distribution of C, N, and O elements, along with the uniform loading of Fe, Cu, and Ce elements onto the carbon aerogel.

### 3.2. Structure and Composition State

#### 3.2.1. XRD Characterization

**Figure 4:**
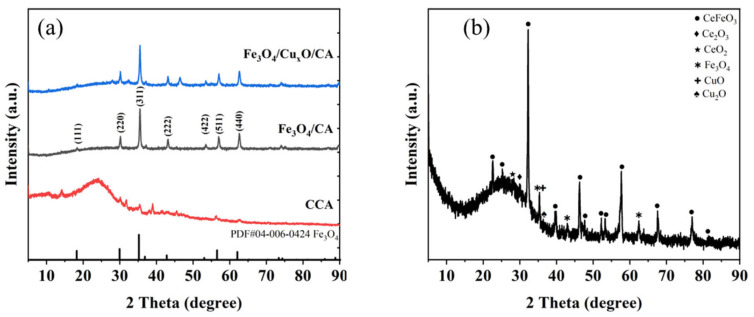
(**a**) XRD pattern of iron and copper metal oxides/carbon aerogel and (**b**) XRD pattern of iron, copper, and cerium metal oxides/carbon aerogel.

As shown in Figure 4, the XRD diffraction patterns of the samples show peaks at 2θ = 18.143°, 29.840°, 35.145°, 36.762°, 42.707°, 52.969°, 56.459°, 61.987°, and 73.300°, corresponding to the (111), (220), (311), (222), (400), (422), (511), (440), and (533) crystal planes of Fe_3_O_4,_ respectively, as referenced to the standard pattern PDF#04-006-0424 (cubic crystal system, space group Fd-3m (227)). The red curve displays a broadened “steamed bun peak” between 15 and 30°, indicative of amorphous substances, primarily attributed to the presence of carbon lacking a long-range ordered structure. In contrast, the blue curve has sharper and clearer diffraction peaks, indicating enhanced crystallinity. The black curve exhibits moderately sharp diffraction peaks with slightly reduced crystallinity compared to the blue curve. The incorporation of cerium during co-precipitation with iron salts, followed by carbonization, leads to the formation of cerium ferrite, a perovskite-type composite oxide.

#### 3.2.2. High-Resolution XPS Spectroscopic Analysis

**Figure 5:**
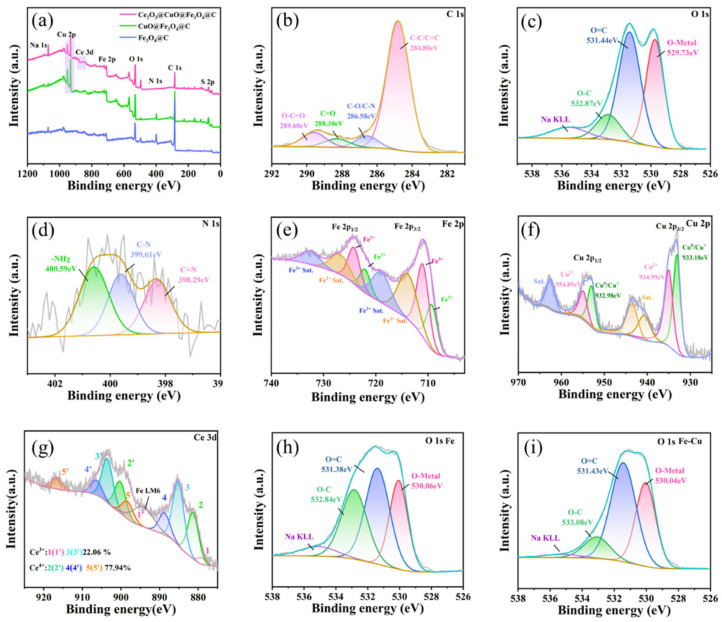
(**a**) The full spectrum; (**b**) the high-resolution spectrum of the C 1s orbital; (**c**) the high-resolution spectrum of the O 1s orbital; (**d**) the high-resolution spectrum of the N 1s orbital; (**e**) the high-resolution spectrum of the Fe 2p orbital; (**f)** the high-resolution spectrum of the Cu 2p orbital, and (**g**) the high-resolution spectrum of the Ce 3d orbital. (**h**) High-resolution spectroscopy of the O 1s orbit in iron materials; (**i**) High-resolution spectra of the O 1s orbitals in iron and copper materials.

The XPS spectra of the materials provide conclusive evidence for determining both the elements and their oxidation states on the material surfaces [20]. Figure 5a shows the full spectra of the three doped samples. It can be seen that the Fe_3_O_4/_CA sample contains elements such as C, O, N, and Fe. Among them, C mainly originates from the aerogel material. Upon Cu incorporation, a diffraction Cu2p peak emerges at approximately 930 eV, indicating the successful loading of CuO. After the loading of Ce_2_O_3_, a peak belonging to Ce3d can be seen between 880 and 920 eV, which also indicates the successful incorporation of Ce_2_O_3_. No new substances are generated, indicating the high purity of the prepared materials [21]. Figure 5b shows the high-resolution spectrum of the C 1s orbital. The characteristic peaks at 284.8 eV, 286.58 eV, 288.30 eV, and 289.60 eV are attributed to C-C/C=C, C-O, C=O, and O-C=O bonds in the aerogel material, respectively, indicating the successful preparation of the gel material and the presence of abundant oxygen-containing functional groups [22]. Figure 5c presents the high-resolution spectrum of the O 1s orbital. For the composite samples, the peak at 529.73 eV is attributed to the metal oxides in the sample, which are speculated to come from Cu-O, Fe-O, and Ce-O bonds. The peak at 531.44 eV is mainly caused by the O=C bond, while that at 532.87 eV is mainly due to the O-C bond in the sample. These functional groups are consistent with those in C 1s, further indicating the abundance of oxygen-containing functional groups in the composite aerogel material [23]. Figure 5d shows the high-resolution spectrum of the N 1s orbital. The peaks at 398.29 eV, 399.61 eV, and 400.59 eV are attributed to C=N, C-N, and -NH_x_ bonds in the aerogel material, respectively. Meanwhile, the signal-to-noise ratio of N1s is relatively large and the content is significantly reduced, which is speculated to be due to the signal not being detected due to the coverage of multiple materials [24]. Figure 5e displays the high-resolution spectrum of Fe 2p, exhibiting a spin–orbit pair splitting into Fe 2p 3/2 and Fe 2p 1/2. Through fitting, it is found that the peaks at 709.65 eV and 723.13 eV are mainly from Fe^2+^, while the peaks at 711.37 eV and 725.01 eV are mainly from Fe^3+^, and those at 715–720 eV and 725–730 eV are satellite peaks of 2+ and 3+. These findings confirm the coexistence of both Fe^2+^ and Fe^3+^ valence states in the composite samples. Combined with the metal oxides in O, it is speculated that Fe_3_O_4_ has been successfully loaded [25]. Figure 5f shows the high-resolution spectrum of Cu 2p, exhibiting the spin–orbit doublet of the Cu 2p 3/2 and Cu 2p 1/2 states. The peaks at 933.10 eV and 932.98 eV are mainly from cuprous species, those at 934.99 eV and 954.89 eV are mainly from Cu^2+^, and those at 942 eV and 963 eV are the characteristic satellite peaks of 2+Cu ions. This indicates that Cu ions in the composite samples mainly exist in the 2+ and 1+ forms. It is speculated that CuO has been successfully loaded onto the sample surface, and a certain reduction has occurred during the process, generating some cuprous species [26]. Figure 5g shows the high-resolution spectrum of the Ce 3d orbital. The peaks at 879.15 eV and 885.14 eV (1 and 3) are attributed to Ce^3+^ in the Ce 3d5/2 orbital, and the peaks at 881.31 eV, 888.75 eV, and 898.28 eV (2, 4, and 5) are attributed to Ce^4+^ in the Ce 3d5/2 orbital. The diffraction peaks at 898.05 eV and 903.53 eV (1′ and 3′) are attributed to the Ce 3d3/2 orbital of Ce3+, while those at 900.21 eV, 906.35 eV, and 916.62 eV (2′, 4′, and 5′) are attributed to the Ce 3d3/2 orbital of Ce^4+^. This indicates that Ce exists as Ce^4+^ and Ce^3+^ in the material. The diffraction peak near 894 mainly comes from the Auger interference peak of the Fe element [27].

#### 3.2.3. Analysis of Specific Surface Area

**Figure 6:**
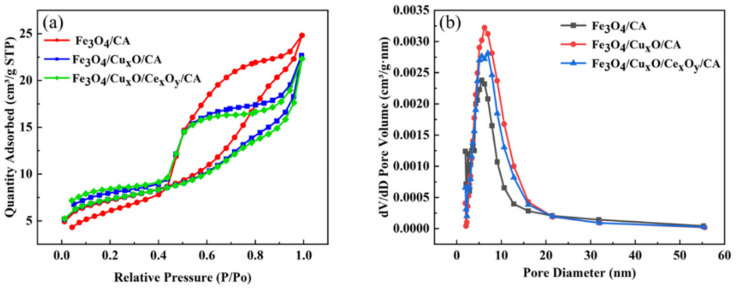
(**a**) The N_2_ adsorption and desorption curves of the material and (**b**) the pore size distribution of the material.

The N_2_ adsorption–desorption isotherm of the material exhibits a typical type IV isotherm, confirming the mesoporous nature of all samples [28]. As shown in Figure 6a, the synthesized materials demonstrate relatively high specific surface areas of 26.67, 26.08, and 26.53 m^2^/g, respectively. The pore size distribution curve indicates that the majority of pores fall within the 0–20 nm range [29]. These structural features, including the substantial surface area and mesoporous material, facilitate increased active site availability and enhanced pollutant adsorption capacity, ultimately contributing to improved catalytic performance [30].

#### 3.2.4. VSM Test Analysis

**Figure 7:**
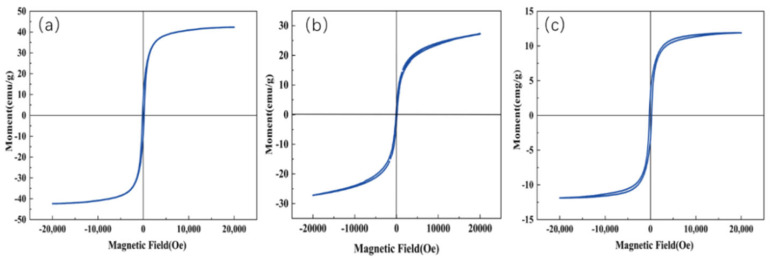
(**a**) The hysteresis curve of the Fe_3_O_4_/CA sample, (**b**) the hysteresis curve of the Fe_3_O_4_/Cu_x_O/CA sample, and (**c**) the hysteresis curve of the Fe_3_O_4_/Cu_x_O /Ce_x_O_y_/CA sample.

As shown in Figure 7, the vibration sample magnetometer (VSM) test results of the sample are presented. From the hysteresis curve in Figure 7a, it can be seen that the saturation magnetization (Ms) of the Fe_3_O_4_/CA sample is 42.26 emu/g, satisfying the requirement for efficient magnetic recovery under an external field [31]. The residual magnetism (Mr) of 8.91 emu/g remains relatively low, facilitating the prompt release of pollutants after magnetic separation and minimizing secondary treatment [32]. The coercive force (Hc) is 185.57 Oe, which belongs to semi-hard magnetic materials and can reduce the risk of demagnetization [33]. Figure 7b displays the hysteresis curve of the Fe_3_O_4_/Cu_x_O/CA sample, with Ms of 27.20 emu/g, meeting the basic requirements for recovery in this trip. The Hc of 108.88 oe indicates weak ferromagnetic behavior, effectively preventing activity loss due to particle agglomeration [34]. Furthermore, the low Mr of 2.43 emu/g significantly minimizes post-reaction particle retention, thereby reducing the potential for separation equipment clogging [35]. Figure 7c shows the hysteresis curve of the Fe_3_O_4_/Cu_x_O/Ce_x_O_y_/CA sample, with Ms being only 11.90 emu/g. It may lead to difficulties in magnetic recovery. The reason for this might be that the tetravalent cerium shifts the Fe^3+^/Fe^2+^ ratio towards the non-magnetic Fe^3+^, weakening the formation of the Fe_3_O_4_ phase [36]. At the same time, the triple non-ferromagnetic components further dilute the magnetism, resulting in the weakest magnetism and a higher Hc of 246.55 Oe [37]. After magnetization, it is not easy to demagnetize, and this is conducive to recovery. The Mr was 3.31 emu/g, suggesting minimal residual magnetism after demagnetization [38].

### 3.3. The Adsorption and Catalytic Properties of Materials

#### 3.3.1. Adsorption Kinetics

To study the adsorption kinetics, the quasi-first-order kinetic, quasi-second-order kinetic, and intra-particle diffusion models were adopted to describe the adsorption efficiency of Fe_3_O_4_/CA for phenol [39]. The corresponding kinetic equations are as follows:

(3) $$\log\left(Q_{e}-Q_{t}\right)=\log Q_{e}-\frac{k_{1}}{2.303}t$$

(4) $$\frac{t}{Q_{t}}=\frac{1}{k_{2}Q_{e}^{2}}+\frac{t}{Q_{e}}$$

(5) $$Q_{t}=k_{p}t^{\frac{1}{2}}+C$$

Among them, Q_e_ (mgg^−1^) and Q_t_ (mgg^−1^) respectively represent the adsorption capacity at equilibrium and time t. k1 (min^−1^), k2 (mgg^−1^min^−1^), and k_p_ (mgg^−1^min^−1/2^) represent quasi-first-order, quasi-second-order, and in-particle diffusion rate coefficients, respectively.

**Figure 8:**
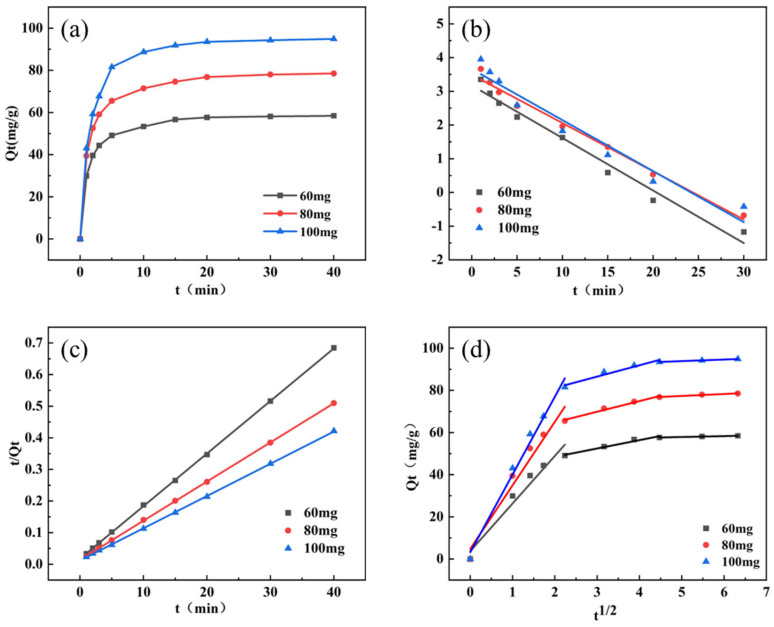
(**a**) The dynamic adsorption curve of phenol by Fe_3_O_4_/CA composite material, (**b**) the quasi-first-order kinetic model fitting of phenol adsorption by the material, (**c**) the quasi-second-order kinetic model fitting of phenol by the material, and (**d**) the intra-particle diffusion kinetic model fitting of phenol by the material. Conditions: The volume of the solution is 40 mL, and the initial pH of the solution is 3.0.

**Figure 9:**
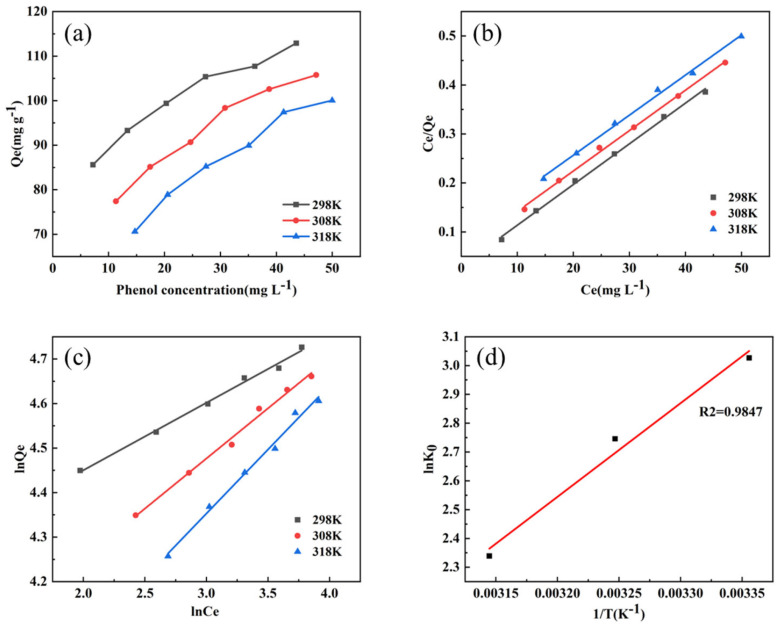
(**a**) The equilibrium adsorption isotherms of phenol by Fe_3_O_4_/CA composites at different temperatures, (**b**) the Langmuir model, (**c**) the Freundlich model, and (**d**) the relationship graph of lnK0 and 1/T in the Gibbs free energy calculation, from which △H^θ^ and △S^θ^ can be calculated, and thus △G^θ^ can be calculated.

As shown in Figure 8a, the adsorption capacity increases rapidly within ten minutes and reaches adsorption equilibrium after 20 min. Comparative analysis of the adsorption curves at different temperatures (Figure 9d) reveals that the time required to reach adsorption equilibrium shortens with rising temperature [40]. The quasi-first-order diagram in Figure 8b assumes that adsorption is controlled by the diffusion steps, whereas the quasi-second-order diagram in Figure 8c indicates that the adsorption rate is jointly determined by both the availability of unoccupied active sites and adsorbate concentration, implicating electron transfer mechanisms [41]. By fitting the dynamic model, it is concluded that the R^2^ of the quasi-second-order model is greater than that of the quasi-first-order model [42]. The intraparticle diffusion kinetics model (Figure 8d) contains three time-varying regions, indicating that the adsorption rate is governed by three stages [43]. The initial stage can be attributed to the diffusion stage of phenol in the boundary layer, while the control factor of the reaction process in the second stage is intraparticle diffusion, and adsorption equilibrium is reached in the third stage [44].

#### 3.3.2. Adsorption Thermodynamics

The Langmuir model is based on the ideal homogeneous monolayer adsorption theory, while the Freundlich equation is based on the assumption of heterogeneous multilayer adsorption [45]. Both can be expressed by the following linear Equations (6) and (7), respectively:

(6) $$\frac{C_{e}}{Q_{e}}=\frac{1}{Q_{m}K_{L}}+\frac{C_{e}}{Q_{m}}$$

(7) $$\ln Q_{e}=\ln K_{F}+\frac{1}{n}\ln C_{e}$$

Among them, K_L_ (L·mg^−1^) and K_F_ (mg^1−n^L^n^g^−1^) are the adsorption equilibrium constants of the Langmuir model and the Freundlich model. A 1/n value between 0 and 1 indicates a favorable adsorption process [46].

As shown in Figure 9, the Langmuir model exhibits higher R^2^ values across various temperatures compared to the Freundlich model, suggesting better conformity with the adsorption data [47]. This observation implies that the adsorption material possesses uniformly distributed active sites with equivalent energy and adsorption affinity [48]. In addition, the maximum adsorption capacity decreases with the increase in temperature, indicating that physical adsorption is involved in the adsorption process [49].

To study the spontaneity of the adsorption process, the Gibbs free energy is calculated using the following formula:

(8) $$△G^{\theta }=RT\ln K_{0}$$

(9) $$△G^{\theta }=△H^{\theta }-T△S^{\theta }$$

(10) $$\ln K_{0}=\frac{△S^{\theta }}{R}-\frac{△H^{\theta }}{RT}$$

Among them, K_0_ (L·mg^−1^) is the equilibrium constant, while R represents the general gas constant (8.314 J·mol^−1^·K^−1^).

Through calculation, it can be found that the △G^θ^ at temperatures of 298 K, 308 K and 318 K are −7.7 KJ mol^−1^,−7.08 KJ mol^−1^ and −6.41 KJ mol^−1^ respectively, all of which are negative values and fall between −20 and 0 KJ mol^−1^, indicating the spontaneous nature of the adsorption process and its dominance by physical adsorption [50]. Additionally, the negative △H^θ^ value further supports the exothermic character of the adsorption process, consistent with the observed decrease in adsorption capacity with increasing temperature [51].

#### 3.3.3. Catalytic Degradation with Metal Oxides/Carbon Aerogels

**Figure 10:**
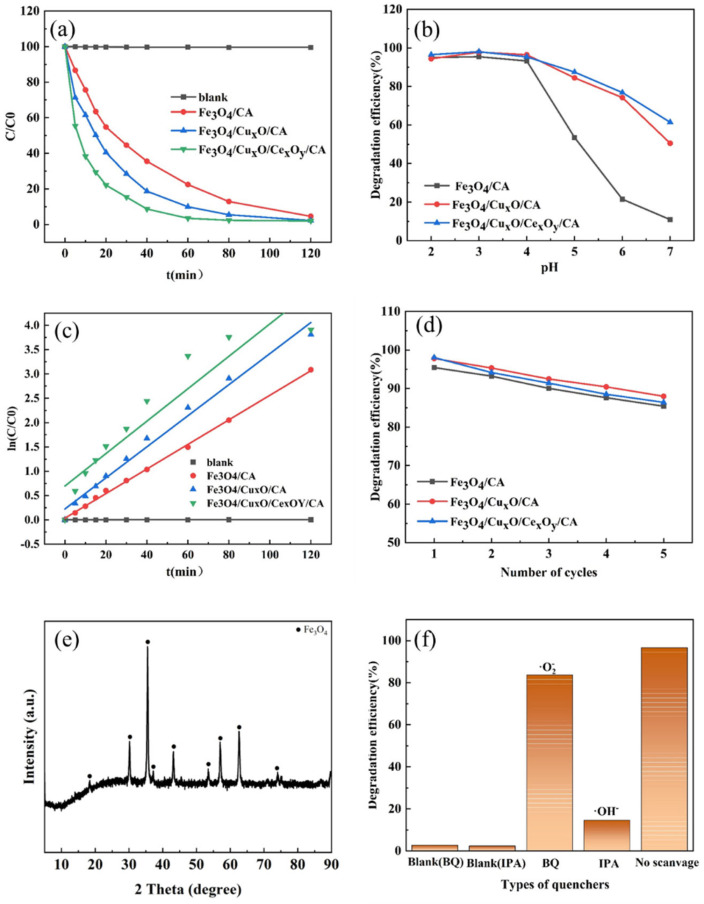
(**a**) The degradation rate of phenol by the material over time, (**b**) the degradation rate of phenol by the material under different pH values, (**c**) the kinetics of phenol degradation, (**d**) the five-cycle phenol experiment using the material, (**e**) XRD data of the material after five cycles, and (**f**) the free radical quenching experiment.

To evaluate the Fenton catalytic oxidation performance of the material for phenol, a series of experiments was conducted. As can be seen from Figure 10a, all three catalysts achieved phenol degradation efficiencies exceeding 95% (95.43, respectively) at pH 3 [52]. Notably, the materials incorporating copper and cerium elements require a shorter time to reach the maximum degradation rates [53]. This enhancement is mainly attributed to the introduction of Cu^+^/Cu^2+^ and Ce^3+^/Ce^4+^, which not only accelerate the Fe^2+^/Fe^3+^ cycle to promote OH^−^ generation but also enable Ce^3+^/Ce^4+^ to participate in Fenton-like reactions, further contributing to ·OH^−^ production through their redox cycling [54]. Furthermore, the incorporation of these multivalent metal ions broadens the operational pH range, as shown in Figure 10b [55]. The material still maintained a certain catalytic degradation capacity for phenol within the pH range of 5–7 [56]. Using Formula (11) as the pseudo-first-order model to fit the degradation kinetics of phenol, where C_0_ is the initial concentration of the solution, C is the concentration at different times, k is the reaction rate constant (min^−1^), and t is the time (min), as shown in Figure 10c. All the data were well fitted by the pseudo-first-order kinetic model.

(11) $$\ln(\frac{C}{C_{0}})=-kt$$

As shown in Figure 10d,e the catalyst demonstrates excellent stability, retaining over 85% of its catalytic degradation capacity after five cycles under optimal pH conditions [57]. Meanwhile, the structure of the crystal did not undergo any significant changes and remained the same as before. The free radical quenching experiment showed that capturing ·OH^−^ reduced the catalytic efficiency by 81.08, suggesting that the catalytic degradation process is mainly related to ·OH^−^ [58].

### 3.4. The Mechanism of Fenton Catalytic Oxidation Degradation of Phenol

**Figure 11:**
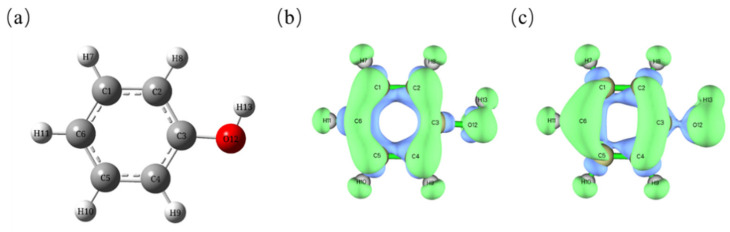
(**a**) shows the phenol molecule model, (**b**) shows the isosurface map of f^0^, and (**c**) shows the isosurface map of f^−^.

The rapid development of computational chemistry has made it possible to predict the reaction sites of relatively large molecules [59]. The Fukui function calculation based on density functional theory (DFT) is a commonly used tool for predicting the region of radical selection reactions [60]. Based on this, we conducted a theoretical prediction of the phenol degradation pathway [61,62]. Figure 11 and Table 1 show the phenol atom and isosurface maps, as well as the Fukui index analysis results of each atom.

The f^+^ in the table is the nucleophilic reaction index, calculated as f^+^(r) = q (N + 1) − q (N), f^−^ is the electrophilic reaction index, calculated as f^−^(r) = q (N) − q (N − 1), f^0^ is the radical reaction index, which is used to predict the site of radical reactions, and Δf indicates the difference between nucleophilic and electrophilic activities. The core mechanism of the Fenton reaction, catalyzing the degradation of phenol, is the free radical chain reaction [63]. Fe^2+^ catalyzes the decomposition of H_2_O_2_ to produce ·OH, which triggers the free radical chain reaction [64]. As can be seen from the Table 1 and Figure 11b, the f^0^ of carbon at positions 1, 2, 4, 5, and 6 is relatively large, and may undergo free radical addition reactions [65]. However, ·OH has strong electrophilicity. It tends to attack the electron-rich region to undergo free radical addition reactions [66]. As can be seen from the Table 1 and Figure 11c, the f^−^ of carbon at positions 1–5 is smaller than that at other positions, that is, the electron cloud density is less than that at the ortho and para positions, generating hydroquinone and o-diphenol [67]. Meanwhile, the electron cloud density at the para position is greater than that at the ortho position, and the amount of o-diphenol generated should be greater than that at the o-diphenol position [68]. Then ·OH continues to attack the benzene ring, causing the C-C bond to break and form aliphatic carboxylic acids (such as maleic acid and oxalic acid) [69]. Subsequently, the carboxylic acids are attacked by ⋅OH and gradually decarboxylated, ultimately generating CO_2_ and H_2_O [70]. Figure 11 shows the prediction graph of the intermediate products of the reaction.

**Figure 12:**
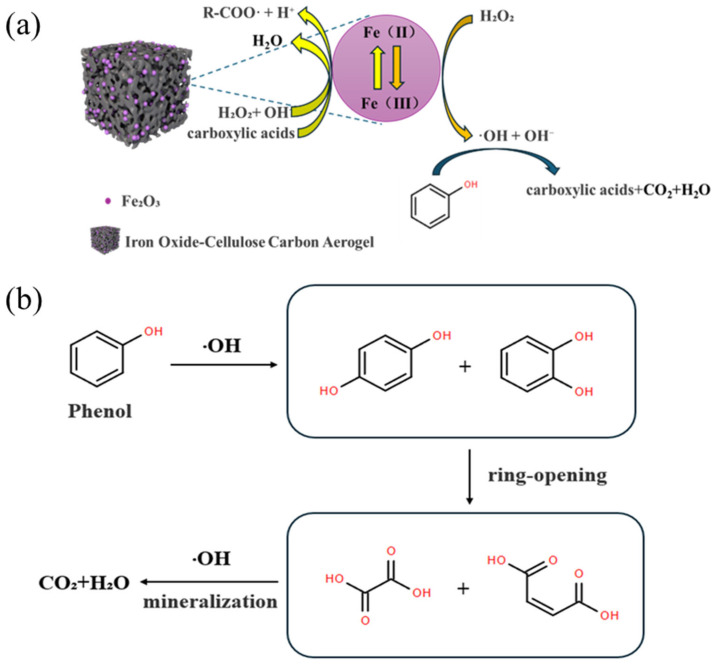
(**a**) A schematic diagram of Fe_3_O_4_ participating in the Fenton catalytic oxidation degradation of phenol and (**b**) the intermediate products of phenol degradation.

Based on the above analysis and existing literature, as shown in Figure 12, the mechanism of phenol degradation via Fenton-like catalytic oxidation employing Fe_3_O_4_/CA can be elucidated as follows: The hydroxyl group (−OH) of phenol combines with Fe^3+^ or the surface of the catalyst through hydrogen bonds, causing the benzene ring to diffuse in the ·OH direction [71]. The ⋅OH attacks the electron-rich sites (ortho/para) of the benzene ring, yielding catechol by ortho attack and hydroquinone by para attack [72]. Subsequently, the intermediate products continue to oxidize, and the benzene ring opens. The C-C bond of catechol/hydroquinone is oxidized and broken by ·OH to form aliphatic carboxylic acids (such as maleic acid and oxalic acid) [73]. Subsequently, the carboxylic acid is mineralized and gradually deacidified by ·OH attack, eventually generating CO_2_ and H_2_O [74]. At the same time, the carboxylic acid also acts as a reducing ligand to reduce Fe^3+^ to Fe^2+^ [75]. The reaction formula is as follows, taking oxalic acid as an example:

(12) $${Fe}^{2+}+H_{2}O_{2}\to {Fe}^{3+}+\cdot OH+OH^{-}$$

(13) $$C_{6}H_{4}{\left(OH\right)}_{2}+\cdot OH\to HOOC-CH_{2}-CH_{2}-COOH$$

(14) $$HOOC-CH_{2}-CH_{2}-COOH+\cdot OH\to {CO}_{2}+H_{2}O$$

(15) $$HOOC-CH_{2}-CH_{2}-COOH+{Fe}^{3+}\to {Fe}^{2+}+{\cdot COO}^{-}+{CO}_{2}+H^{+}$$

**Figure 13:**
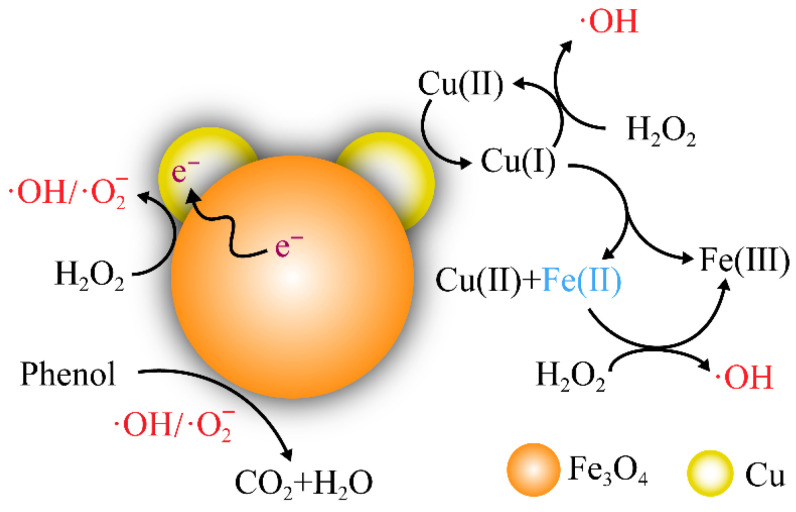
A schematic diagram of the degradation of phenol in the Cu and Fe system.

As shown in Figure 13, the addition of the transition metal Cu promotes the cycling of Fe^3+^/Fe^2+^, accelerates the charge transfer process, and promotes the generation of ∙OH. At the same time, Cu^+^/Cu^2+^ can directly react with H_2_O_2_ to produce ∙OH [76], thereby enhancing the degradation efficiency of phenol. The introduction of Ce can cause an increase in Cu(I) and unpaired electrons, which is beneficial for electron transfer and the regeneration of active sites. Moreover, Ce^3+^/Ce^4+^ can also, theoretically, catalyze the decomposition of H_2_O_2_ to generate ∙OH. 

## 4. Conclusions

The iron oxide-loaded carbon aerogel was synthesized via polyethyleneimine-assisted crosslinking and subsequently applied in the Fenton reaction for phenol degradation. The synthesized material features a high specific surface area and porous structure, offering abundant active sites to promote H_2_O_2_ adsorption and ·OH generation. The Fe^3+^ incorporated in the iron oxide carbon aerogel undergoes reduction to Fe^2+^ through either carbon matrix-mediated electron transfer or surface-adsorbed organic intermediates (such as short-chain carboxylic acids), thereby minimizing iron sludge formation and extending the catalyst’s cycling performance. The iron oxide particles loaded on a carbon skeleton can inhibit the dissolution of active components and prevent catalyst deactivation caused by Fe^3+^ deposition. The composite catalyst synthesized by simultaneously introducing Cu^+^/Cu^2+^and Ce^3+^/Ce^4+^ promotes Fe^2+^/Fe^3+^ cycling, leading to an accelerated rate of phenol degradation and a broadened operational pH range. Remarkably, the catalyst maintains its degradation efficiency without significant loss after five reaction cycles, demonstrating excellent catalytic effect and stability.

## Figures and Tables

**Table 1:** Fukui index of each atom in phenol.

Atom	q(N)	q(N + 1)	q(N − 1)	f^−^	f^+^	f^0^	Δf
1(C)	−0.0392	−0.1684	0.0357	0.075	0.1292	0.1021	0.0542
2(C)	−0.0709	−0.1917	0.0116	0.0825	0.1207	0.1016	0.0382
3(C)	0.0738	0.0212	0.1736	0.0998	0.0526	0.0762	−0.0472
4(C)	−0.0578	−0.1832	0.0333	0.0911	0.1254	0.1083	0.0343
5(C)	−0.0367	−0.1632	0.0295	0.0661	0.1265	0.0963	0.0604
6(C)	−0.0565	−0.1176	0.0875	0.144	0.0611	0.1026	−0.083
7(H)	0.0417	−0.0254	0.0888	0.0471	0.0671	0.0571	0.02
8(H)	0.0359	−0.0278	0.0828	0.0469	0.0637	0.0553	0.0168
9(H)	0.0445	−0.0208	0.0928	0.0483	0.0653	0.0568	0.0169
10(H)	0.0423	−0.0238	0.0879	0.0456	0.0661	0.0558	0.0205
11(H)	0.0386	−0.0077	0.0973	0.0587	0.0463	0.0525	−0.0124
12(O)	−0.1886	−0.2335	−0.0451	0.1435	0.0448	0.0942	−0.0987
13(H)	0.1728	0.1418	0.2241	0.0512	0.0311	0.0412	−0.0202

## Data Availability

The data is unavailable due to privacy and ethical restrictions.

## Abbreviations

The following abbreviations are used in this manuscript:

BQ	Benzoquinone
IPA	Isopropanol

## References

[1] Alinasab M., Navidjouy N., Alizadeh S., Rahimnejad M. (2025). Bio-Electro-Fenton system assisted with metal-organic framework for degradation of bis-phenol S in wastewater as an emerging contaminant. Sci. Rep..

[2] da Silva Aires F.I., Dari D.N., Freitas I.S., da Silva J.L., de Matos Filho J.R., dos Santos K.M., de Castro Bizerra V., Sales M.B., de Souza Magalhães F.L., da Silva Sousa P. (2024). Advanced and prospects in phenol wastewater treatment technologies: Unveiling opportunities and trends. Discov. Water.

[3] Liu X., Xu H.H., Fu X., Chen J.Y. (2024). Steam-assisted synthesis of hectorite loaded with Fe_2_O_3_ and its catalytic Fenton degradation of phenol. Catalysts.

[4] Kanakaraju D., Glass B.D., Goh P.S. (2025). Advanced oxidation process-mediated removal of pharmaceuticals from water: A review of recent advances. Environ. Sci. Pollut. Res..

[5] Lin Y.J., Dai Y.Z., Zhang L., Wu Q. (2022). Efficient degradation of phenol in aqueous solution by Fe^2+^/H_2_O_2_/CaO_2_ system. Environ. Technol. Innov..

[6] Ribeiro J.P., Marques C.C., Neves M.C., Gomes H.G.M.F., Sarinho L., Tarelho L.A.C., Nunes M.I. (2025). Catalytic performance of thermochemically treated sludge generated in the Fenton process for AOX degradation. J. Ind. Eng. Chem..

[7] Raksha C.H., Yogeesh M.P., Shetty N.S. (2025). Recent advances in the synthesis of polymer supported catalysts: A review. Discov. Appl. Sci..

[8] Bouzayani B., Sanromán M.Á. (2024). Polymer-supported heterogeneous Fenton catalysts for the environmental remediation of wastewater. Molecules.

[9] Feng J., Zhang Y. (2023). Ascorbic acid enhanced CuFe2O4-catalyzed heterogeneous photo-Fenton-like degradation of phenol. J. Environ. Chem. Eng..

[10] Xia Q., Zhang D., Yao Z., Jiang Z. (2022). Revealing the enhancing mechanisms of Fe–Cu bimetallic catalysts for the Fenton-like degradation of phenol. Chemosphere.

[11] Ke P., Zeng D., Wang R., Cui J., Li X., Fu Y. (2022). Magnetic carbon microspheres as a reusable catalyst in heterogeneous Fenton system for the efficient degradation of phenol in wastewater. Colloids Surf. A.

[12] Ren J.X., Chen S.P., Li D.L., Wang M.L., Zhu J.L., Zhong G.J., Huang H.D., Li Z.M. (2024). Hierarchically porous cellulose-based carbon aerogels with N-doped skeletons and encapsulated iron-based catalysts for efficient tetracycline catalytic degradation. Int. J. Biol. Macromol..

[13] Stark F.W., Thue P.S., Missio A.L., Machado F.M., Delucis R.d.A., Andreazza R. (2025). Cellulose-based aerogels for environmentally sustainable applications: A review of the production, modification, and sorption of environmental contaminants. Polymers.

[14] Liu F.Q., Fan M.J., Liu X., Chen J.Y. (2024). One-pot synthesis of cellulose-based carbon aerogel loaded with TiO_2_ and g-C_3_N_4_ and its photocatalytic degradation of Rhodamine B. Nanomaterials.

[15] Liu M.J., Cai S.Y., Srinivasakannan C., Xue G., Wang L., Wang Y.P., Duan X.H. (2025). In-situ growth of metal–organic framework on ionic cross-linking cellulose aerogel for adsorption and Fenton degradation of tetracycline. Sep. Purif. Technol..

[16] Xie L.C., Zhang Z.C., He Y.C., Jiang Y. (2024). Preparation of polyvinyl alcohol-chitosan nanocellulose-biochar nanosilver composite hydrogel and its antibacterial property and dye removal capacity. Processes.

[17] Zhao Q., Yang J.W., Xia J.Y., Zhao G.T., Yang Y.D., Zhang Z.W., Li J., Wei F., Song W.G. (2025). Biomass cellulose-derived carbon aerogel supported magnetite-copper bimetallic heterogeneous Fenton-like catalyst towards the boosting redox cycle of ≡Fe(III)/≡Fe(II). Nanomaterials.

[18] Zhong J.L., Mao X.F., Wang G.Y., Li H., Li J.F., Qu S.J., Zhao J.W. (2024). Synthesis of Cu/Mn/Ce polymetallic oxide catalysts and catalytic ozone treatment of wastewater. RSC Adv..

[19] Zhu J.L., Wang M.L., Shi S.C., Ren J.X., Huang H.D., Lin W., Li Z.M. (2022). In-situ constructing robust cellulose nanocomposite hydrogel network with well-dispersed dual catalysts for the efficient, stable and recyclable photo-Fenton degradation. Cellulose.

[20] Lopinski G.P., Kodra O., Kunc F., Kennedy D.C., Couillard M., Johnston L.J. (2025). X-ray photoelectron spectroscopy of metal oxide nanoparticles: Chemical composition, oxidation state and functional group content. Nanoscale Adv..

[21] Yang F., Hao D.D., Wu M.M., Fu B., Zhang X.F. (2024). Amino-functionalized metal-organic framework-mediated cellulose aerogels for efficient Cr(VI) reduction. Polymers.

[22] Camparotto N.G., Neves T.d.F., Mastelaro V.R., Prediger P. (2023). Hydrophobization of aerogels based on chitosan, nanocellulose and tannic acid: Improvements on the aerogel features and the adsorption of contaminants in water. Environ. Res..

[23] Zhu J.L., Chen S.P., Lin W., Huang H.D., Li Z.M. (2023). Cellulose mineralization with in-situ synthesized amorphous titanium dioxide for enhanced adsorption and auto-accelerating photocatalysis on water pollutant. Chem. Eng. J..

[24] Zhu R.L., Zhu Y.P., Xian H.Y., Yan L.X., Fu H.Y., Zhu G.Q., Xi Y.F., Zhu J.X., He H.P. (2020). CNTs/ferrihydrite as a highly efficient heterogeneous Fenton catalyst for the degradation of bisphenol A: The important role of CNTs in accelerating Fe(III)/Fe(II) cycling. Appl. Catal. B Environ..

[25] Li G.Y., Liu W.F., Gao S.J., Lu H.Y., Fu D.J., Wang M.L., Liu X.G. (2024). MXene-based composite aerogels with bifunctional ferrous ions for the efficient degradation of phenol from wastewater. Chemosphere.

[26] Li J.W., Wei Y.F., Liu Q., Guan H.H., Jiang C.C., Sun X.H. (2025). Heterogeneous Fenton-like CuO-CoOx/SBA-15 catalyst for organic pollutant degradation: Synthesis, performance, and mechanism. Front. Chem..

[27] Melnikova N., Malygina D., Korokin V., Al-Azzawi H., Zdorova D., Mokshin E., Liyaskina E., Kurgaeva I., Revin V. (2023). Synthesis of cerium oxide nanoparticles in a bacterial nanocellulose matrix and the study of their oxidizing and reducing properties. Molecules.

[28] Guan Z.L., Wang Y.D., Wang Z., Hong Y., Liu S.L., Luo H.W., Liu X.L., Su B.L. (2024). The synthesis, characteristics, and application of hierarchical porous materials in carbon dioxide reduction reactions. Catalysts.

[29] Zhao J.X., Yuan X.S., Wu X.X., Liu L., Guo H.Y., Xu K.M., Zhang L.P., Du G.B. (2023). Preparation of nanocellulose-based aerogel and its research progress in wastewater treatment. Molecules.

[30] Kuang J., Cai T.M., Dai J.B., Yao L.H., Liu F.F., Liu Y., Shu J.C., Fan J.P., Peng H.L. (2023). High strength chitin/chitosan-based aerogel with 3D hierarchically macro-meso-microporous structure for high-efficiency adsorption of Cu(II) ions and Congo red. Int. J. Biol. Macromol..

[31] Mao Y.B., Gupta S.K. (2022). Metal oxide nanomaterials: From fundamentals to applications. Nanomaterials.

[32] Petrinic I., Stergar J., Bukšek H., Drofenik M., Gyergyek S., Hélix-Nielsen C., Ban I. (2021). Superparamagnetic Fe_3_O_4_@CA nanoparticles and their potential as draw solution agents in forward osmosis. Nanomaterials.

[33] Rajan A., Sharma M., Sahu N.K. (2020). Assessing magnetic and inductive thermal properties of various surfactants functionalised Fe_3_O_4_ nanoparticles for hyperthermia. Sci. Rep..

[34] Alani O.A., Ari H.A., Alani S.O., Offiong N.-A.O., Feng W. (2021). Visible-light-driven bio-templated magnetic copper oxide composite for heterogeneous photo-Fenton degradation of tetracycline. Water.

[35] Sabzi Dizajyekan B., Jafari A., Vafaie-Sefti M., Saber R., Fakhroueian Z. (2024). Preparation of stable colloidal dispersion of surface modified Fe_3_O_4_ nanoparticles for magnetic heating applications. Sci. Rep..

[36] Liu M.Y., Ye Y.Y., Ye J.M., Gao T., Wang D.H., Chen G., Song Z.J. (2023). Recent advances of magnetite (Fe_3_O_4_)-based magnetic materials in catalytic applications. Magnetochemistry.

[37] Xu J.C., Su H., Kumar R., Luo S.S., Nie Z.Y., Wang A., Du F., Li R., Smidman M., Yuan H.Q. (2021). Ce-site dilution in the ferromagnetic kondo lattice CeRh_6_Ge_4_. Chin. Phys. Lett..

[38] Horsley E., Rao X., Yi S.B., Kim Y.J. (2022). Magnetic dilution of a honeycomb lattice XY magnet CoTiO_3_. J. Phys. Condens. Mat..

[39] Allahkarami E., Dehghan Monfared A., Silva L.F.O., Dotto G.L. (2022). Lead ferrite-activated carbon magnetic composite for efficient removal of phenol from aqueous solutions: Synthesis, characterization, and adsorption studies. Sci. Rep..

[40] Si R.R., Pu J.W., Luo H.G., Wu C.J., Duan G.G. (2022). Nanocellulose-based adsorbents for heavy metal ion. Polymers.

[41] Wang B.X., Xu B.B., Chen G.F., Wang C.Z., Liu Y., Bai Y., Li M.G., Peng L.G. (2025). Preparation of polypyrrole/montmorillonite/polypropylene composite membranes and investigation of their adsorption performance for methyl orange and Pb^2+^. Polymers.

[42] Meng W.Y., Wang S.J., Lv H.F., Wang Z.X., Han X.W., Zhou Z.J., Pu J.W. (2022). Porous cellulose nanofiber (CNF)-based aerogel with the loading of zeolitic imidazolate frameworks-8 (ZIF-8) for Cu (II) removal from wastewater. BioResources.

[43] Hasani N., Selimi T., Mele A., Thaçi V., Halili J., Berisha A., Sadiku M. (2022). Theoretical, equilibrium, kinetics and thermodynamic investigations of methylene blue adsorption onto lignite coal. Molecules.

[44] Liu P.P., Zheng C.L., Yao Z., Zhang F. (2024). A biomimetic lignocellulose aerogel-based membrane for efficient phenol extraction from water. Gels.

[45] Ehiomogue P., Ahuchaogu I.I., Ahaneku I.E. (2021). Review of adsorption isotherms models. Acta Tech. Corviniensis-Bull. Eng..

[46] Dal M.C. (2024). Modeling of the linear equations of langmuir isotherm in the adsorption of Cd (II) Ion with siirt kurtalan koçpinar clay. Türk Doğa ve Fen Dergisi.

[47] Cho E.J., Lee C.G., Park S.J. (2024). Adsorption of phenol on kenaf-derived biochar: Studies on physicochemical and adsorption characteristics and mechanism. Biomass Convers. Biorefin..

[48] Dehmani Y., Sellaoui L., Alghamdi Y., Lainé J., Badawi M., Amhoud A., Bonilla-Petriciolet A., Lamhasni T., Abouarnadasse S. (2020). Kinetic, thermodynamic and mechanism study of the adsorption of phenol on Moroccan clay. J. Mol. Liq..

[49] Zhang J.C., Qin L., Yang Y.Z., Liu X.G. (2021). Porous carbon nanospheres aerogel based molecularly imprinted polymer for efficient phenol adsorption and removal from wastewater. Sep. Purif. Technol..

[50] Salvestrini S., Fenti A., Chianese S., Iovino P., Musmarra D. (2020). Diclofenac sorption from synthetic water: Kinetic and thermodynamic analysis. J. Environ. Chem. Eng..

[51] Edet U.A., Ifelebuegu A.O. (2020). Kinetics, isotherms, and thermodynamic modeling of the adsorption of phosphates from model wastewater using recycled brick waste. Processes.

[52] Xu Y.L., Peng Q., Yu Y.L., Li Z.F., Liu Y.M., Qin Z.J., Fan X.F. (2025). Facile electrophoretically deposited of iron/carbon nanotube-based electro-Fenton cathode for high-performance organics degradation. J. Environ. Chem. Eng..

[53] Eltaweil A.S., Galal A.M., Abd El-Monaem E.M., Al Harby N., Batouti M.E. (2024). Enhanced Fenton degradation of tetracycline over cerium-doped MIL88-A/g-C_3_N_4_: Catalytic performance and mechanism. Nanomaterials.

[54] Peera S.G., Kim S.W. (2025). Rare earth Ce/CeO_2_ electrocatalysts: Role of high electronic spin state of Ce and Ce^3+^/Ce^4+^ redox couple on oxygen reduction reaction. Nanomaterials.

[55] Wei J.H., Yuan M., Wang S.T., Wang X.H., An N., Lv G.P., Wu L.N. (2023). Recent advances in metal organic frameworks for the catalytic degradation of organic pollutants. Collagen Leather.

[56] Zhang H.N., Tian L.H., Zhang Z.Q., Han J.P., Wu Z.G., Wei Z.Q., Wang S.F., Cao Y., Zhang S., Zhang Y. (2024). Effective degradation of phenol by in-situ photocatalytic-Fenton-like technology with BiVO_4_/Bi_2_WO_6_/Ti_3_C_2_ QDs. Surf. Interf..

[57] Yang Z.C., Yin Y.Y., Liang M.Y., Fu W.Y., Zhang J.H., Liu F.Z., Zhang W., Pan B.C. (2025). Incidental iron oxide nanoclusters drive confined Fenton-like detoxification of solid wastes towards sustainable resource recovery. Nat. Commun..

[58] Yin X.Z., Yin H.Q., Wang R.J., Wang J.N., Li A.M. (2024). Novel Fenton-like catalyst HKUST-1(Cu)/MoS_2_-3-C with non-equilibrium-state surface for selective degradation of phenolic contaminants: Synergistic effects of σ-Cu-ligand and ≡Mo–OOSO_3_− complex. Water.

[59] Gu S.J., Cui J.Y., Liu F.Q., Chen J.Y. (2023). Biochar loaded with cobalt ferrate activated persulfate to degrade naphthalene. RSC Adv..

[60] Xin L., Hu J.W., Xiang Y.Q., Li C.F., Fu L.Y., Li Q.H., Wei X.H. (2021). Carbon-based nanocomposites as Fenton-like catalysts in wastewater treatment applications: A review. Materials.

[61] Ye F., Jin X., Chen Z. (2025). Biomass-derived in-situ carbonized ferrous sulfide for Fenton oxidation of phenol in wastewater: Mechanisms, pathways, and applications. J. Colloid Interface Sci..

[62] Liu Z., Zhang Y., Lee J., Xing L. (2024). A review of application mechanism and research progress of Fe/montmorillonite-based catalysts in heterogeneous Fenton reactions. J. Environ. Chem. Eng..

[63] Yang R.S., Liu Q., Liu Z., Wang J.X., Zhang A.N., Liu Y.J. (2025). Insights into the Fenton-like degradation efficiency and mechanism of phenolic pollutants by Cu-doped sludge-based biochar catalyst. Chem. Eng. Sci..

[64] Tsuneda T. (2020). Fenton reaction mechanism generating no OH radicals in Nafion membrane decomposition. Sci. Rep..

[65] Iii R.C., Lujan B., Martinez A., Manasi R., DeBow J.D., Kou K.G.M. (2023). A Fenton approach to aromatic radical cations and diarylmethane synthesis. J. Org. Chem..

[66] Thomas N., Dionysiou D.D., Pillai S.C. (2021). Heterogeneous Fenton catalysts: A review of recent advances. J. Hazard. Mater..

[67] Meng Y., Liu Y.Q., Wang C., Si Y., Wang Y.J., Xia W.Q., Liu T., Cao X., Guo Z.Y., Chen J.J. (2024). Nanoconfinement steers nonradical pathway transition in single atom Fenton-like catalysis for improving oxidant utilization. Nat. Commun..

[68] Gonfa M.T., Shen S., Chen L., Yin S.F. (2025). Selective hydroxylation of benzene via enhanced generation and utilization of hydroxyl radicals with CuZnSbO photocatalyst. Colloid. Surf. A.

[69] Walling S.A., Um W., Corkhill C.L., Hyatt N.C. (2021). Fenton and Fenton-like wet oxidation for degradation and destruction of organic radioactive wastes. npj Mater. Degrad..

[70] Cheng P., Mailhot G., Sarakha M., Voyard G., Scheres Firak D., Schaefer T., Herrmann H., Brigante M. (2025). The effect of amino acids on the Fenton and photo-Fenton reactions in cloud water: Unraveling the dual role of glutamic acid. EGUsphere.

[71] Wang X.P., Liu W., Qin J.Y., Lei L.C. (2020). Improvement of H_2_O_2_ utilization by the persistent heterogeneous Fenton reaction with the Fe_3_O_4_-zeolite-cyclodextrin composite. Ind. Eng. Chem. Res..

[72] Pourshaban-Mazandarani M., Ahmadian M., Nasiri A., Poormohammadi A. (2023). CuCoFe_2_O_4_@ AC magnetic nanocomposite as a novel heterogeneous Fenton-like nanocatalyst for Ciprofloxacin degradation from aqueous solutions. Appl. Water Sci..

[73] Zhang X., Tang J.J., Wang L.L., Wang C., Chen L., Chen X.Q., Qian J.S., Pan B.C. (2024). Nanoconfinement-triggered oligomerization pathway for efficient removal of phenolic pollutants via a Fenton-like reaction. Nat. Commun..

[74] Peng L.J., Duan X.G., Shang Y.N., Gao B.Y., Xu X. (2021). Engineered carbon supported single iron atom sites and iron clusters from Fe-rich Enteromorpha for Fenton-like reactions via nonradical pathways. Appl. Catal. B Environ..

[75] Ma J.Q., Xu L.L., Shen C.S., Hu C., Liu W.P., Wen Y.Z. (2018). Fe-N-graphene wrapped Al_2_O_3_/Pentlandite from microalgae: High Fenton catalytic efficiency from enhanced Fe^3+^ reduction. Environ. Sci. Technol..

[76] Zhang N., Yi Y., Lian J., Fang Z. (2020). Effects of Ce doping on the Fenton-like reactivity of Cu-based catalyst to the fluconazole. Chem. Eng. J..

